# Mental Health Needs in Patients With Communication Deficits and Chronic Health Needs: A Case of a Teenager With Goldenhar Syndrome

**DOI:** 10.7759/cureus.91771

**Published:** 2025-09-07

**Authors:** Bertha Beltran-Regalado, Roshan M Panda, Arianna Gregg, Mackenzie Montero, Jose Cucalon Calderon

**Affiliations:** 1 Pediatrics and Child Health, University of Nevada Reno School of Medicine, Reno, USA; 2 Pediatrics, University of Nevada Reno School of Medicine, Reno, USA

**Keywords:** adjustment disorder, child and adolescent psychiatry, chronic health needs, communication deficits, congenital disease, goldenhar syndrome, mental health, nonverbal patient, psychopathology

## Abstract

Goldenhar syndrome, a rare congenital condition, often presents with physical anomalies due to abnormal development of the first and second branchial arches, leading to facial and auricular malformations. However, the mental health challenges associated with this syndrome are often overlooked. This report describes the case of a 14-year-old Latino/Hispanic male child with Goldenhar syndrome and limited verbal communication, highlighting the complexities of managing both physical and psychological aspects of care after establishing follow-up with a pediatrician. The patient demonstrated noncompliance with recommendations and presented with low motivation and symptoms consistent with adjustment disorder and possible low mood, despite repeated normal Patient Health Questionnaire scores. This case underscores the challenges of addressing mental health needs in the setting of communication deficits and limited family support, while emphasizing the lack of data to guide management in Goldenhar syndrome. It highlights the importance of regular mental health screening and the need for providers to recognize the unique challenges of patients with communication deficits by remaining attentive to nonverbal indicators of distress. Ultimately, this case demonstrates that medical care professionals must remain aware of the mental health difficulties faced by children with chronic health conditions and communication deficits to ensure comprehensive, holistic care.

## Introduction

Goldenhar syndrome is a congenital condition that exhibits significant phenotypic and etiological diversity. It primarily impacts structures derived from the first and second pharyngeal arches, including the ears, eyes, mandible, and cervical spine. Furthermore, individuals with this syndrome may also manifest other significant anomalies, including congenital heart defects [1].

Individuals diagnosed with Goldenhar syndrome encounter ongoing challenges throughout their lives as a result of their chronic health condition; however, descriptions of psychopathological disorders within Goldenhar's syndrome are infrequently reported in the literature. Based on available data, mental health diagnoses are documented in approximately one-quarter of cases involving children with this condition [2]. Moreover, findings from the Medical Expenditure Panel Survey data spanning from 2008 to 2013 revealed that children with at least one chronic physical condition had a 62% higher likelihood of experiencing a mental health disorder compared to children without chronic physical conditions [3].

Among the symptoms listed in the literature, we find that as the condition progresses, there is a notable worsening of the psychopathological symptoms, such as delayed motor skill development and speech delay during early childhood. In addition, the psychopathological profile of the condition becomes more complex with the potential inclusion of affective disorders, such as the depressive-dysphoric type and psychotic disorders, leading to heightened emotional and personality changes [2].

Difficulties in the recognition and treatment of psychopathology in patients with Goldenhar syndrome can lead to difficulties in meeting the mental health needs of children, including early detection, family support, and prompt and timely treatment. In this case report, we highlight how these could have led to prompt care, earlier agreement on continued necessary care for this child, decreased non-compliance with appointments and plan of care, and offered better patient and family support options.

## Case presentation

A 14-year-old Latino/Hispanic male child presented to a new primary care physician in April 2019, after being seen as a new patient with a recent respiratory illness that required an ICU stay. The child was already diagnosed with Goldenhar syndrome; the earliest record that includes his Goldenhar diagnosis was when the patient was five years old. Coordination with plastic surgery, ENT, and home medicine was ongoing and had already been established.

The physical examination revealed thin body habitus, poor nutrition, facial tutors, left microtia, and bilateral keloids along the jawline. A tracheostomy and a G-button were in place as the patient continued to require them. The patient was largely nonverbal and communicated through physical gestures, such as nodding and allowing the parent to state his desires. There was no disclosure of the patient's ability to use sign language; the parent was the main source of information and communication. The patient had already undergone a tracheostomy, gastrostomy, tonsillectomy, adenoidectomy, bronchoscopy, multiple mandibular surgeries and dental surgeries, video fluoroscopic swallow study, and bilateral orchiopexy. During this appointment, the parent and patient expressed concerns about continuing with further reconstruction. On subsequent follow-up appointments, the parent and child expressed persistent concerns about facial reconstruction and a reluctance to undergo additional procedures, with the parent reporting on the patient's fatigue from medical interventions, travel, and school absences.

Later appointments in 2019 and onward revealed evolving challenges. The patient had been experiencing dysphagia and drooling and was subsequently referred to speech therapy for an evaluation. They determined he was at risk for aspiration and recommended he remain NPO (nil per os; nothing by mouth) and use a peg tube for feeding, while they performed studies and did therapy; these recommendations were ignored, and regular oral feedings were started against advice, which led to the patient experiencing difficulty swallowing and choking. This prompted re-evaluation by speech therapy.

Compliance with medical recommendations varied, including non-compliance with the recommendations from speech therapy, difficulty attending appointments, and refusal to change or close trachea access as recommended by speech therapy if attempting to feed orally with ongoing concerns for aspiration and weight loss. Additionally, concerns about facial appearance and scarring persisted amid continued scheduled facial surgeries. Even with counseling and ongoing recommendations of increased pulmonary risks, the patient continuously refused Influenza and COVID-19 vaccination yearly, without the parent or the patient offering room to help assess causes for vaccination refusal.

The patient consistently scored zero on Patient Health Questionnaire-2 screenings over four years of documented office visits. Given the reported difficulties and lack of care agreement, lack of compliance with recommended therapy, and low mood and suffering expressed by the parent, concerns for depression and adjustment disorder, and need for treatment were offered, as the patient was at higher risk due to communication deficits and chronic complex health needs. However, an agreement on recommendations for counseling and psychotherapy was not reached. Other factors considered included isolation due to the COVID-19 pandemic, and there was ongoing concern that the patient's noncompliance with continued care and support likely stemmed from an adjustment disorder. At this time, the patient transitioned to adult care with a documented continued care plan.

## Discussion

Goldenhar syndrome is a rare congenital disease, affecting one in every 3,000-5,000 live births. It originates from the abnormal development of the first and second branchial arches and often presents as a triad of vertebral anomalies, mandibular hypoplasia, and ocular and auricular malformations [4]. These malformations can include hemifacial microsomia, facial asymmetry, preauricular skin tags, and epibulbar dermoids. Other craniofacial abnormalities include malformation of the mandible, temporal bone, zygoma, outer ear, and facial nerve [5].

**Figure 1:**
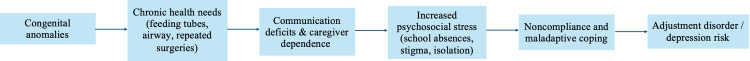
Proposed model of psychopathology risk in Goldenhar syndrome This model illustrates how congenital anomalies in Goldenhar syndrome lead to chronic health needs, which in turn contribute to communication barriers and caregiver dependence. These factors amplify psychosocial stress and can result in maladaptive coping behaviors, ultimately increasing the risk of adjustment disorder and depression [8]. Image Credit: Authors.

It is likely that in this patient population, with chronic health needs and communication deficits, there may be a higher risk for increased mental health needs (Figure 1). In a study conducted by Schuchard et al., findings suggested that the negative impacts of chronic conditions on children and adolescents’ well-being may be primarily credited to an increased risk for continuous emotional distress [6]. Hitchen et al. emphasize the association between repeated conference attendance for parents and children suffering rare syndromes and increased self-efficacy, leading to an overall improved quality of life [7]. Children with rare conditions mustn't be isolated because of this. Having a strong support system can help ease the emotional stress. Therefore, members of the healthcare team must be aware of the emotional toll that chronic conditions can have on patients and their families, so they can provide adequate resources and educational support for family members of individuals with chronic conditions.

Analyzing the mental health status of an individual, however, can become complicated when their verbal communication is limited. The case of our young male patient with Goldenhar syndrome, marked by limited verbal skills and symptoms indicative of an adjustment disorder, presents distinct challenges and insights into the treatment and understanding of this complex condition. Adjustment disorder is characterized as a maladaptive emotional and/or behavioral response to an identifiable psychosocial stressor, with symptoms that are disproportionate to socially or culturally expected reactions to the stressor, resulting in pronounced distress and impairment in daily functioning [9].

Our patient demonstrated maladaptive responses brought about by his chronic condition by refusing to comply with speech therapy recommendations and frequently missing medical appointments. According to the patient's mother, he showed reluctance toward medical procedures, the associated travel, and the resultant school absences. Additionally, he resisted feeding during the day and declined counseling when suggested. All of these are indicative of behaviors that would, in turn, lead to concern for increased distress that can continue or worsen the patient’s emotional well-being and functional abilities, as is seen in individuals dealing with adjustment disorder.

It has been identified that adjustment disorder stressor events may include experiencing a personal illness [8]. This stressor can be particularly complicated in settings where communication deficits are present, and healthcare providers must be aware of both these complications and how they intersect. There is clear evidence, for example, that people with communication disorders experience significantly poorer health outcomes than those without these disorders [10]. The lack of compliance and engagement with counseling seen in our patient, likely stemming from adjustment disorder, could further fuel a less-than-ideal health outcome. As healthcare providers, these communication barriers must be addressed to provide adequate healthcare.

Although symptoms of physical health conditions and developmental disorders can last a lifetime, it is possible to reduce the adverse effects on life satisfaction by incorporating interventions that prevent and treat comorbid mental health problems, such as regular screening for depression, especially among children with chronic conditions [6]. The United States Preventive Services Task Force recommends that routine depression screening start at 12 years of age in primary care settings, as a lack of depression diagnosis can harm the developmental course of those who are affected [11]. Therefore, the healthcare team treating individuals with Goldenhar syndrome should be especially aware of the importance of assessing their mental health, as their chronic condition could put them at an increased risk of acquiring comorbid mental health conditions. In our particular case, we encountered the challenge of assessing mental well-being due to the limited verbal communication our patient possessed. We also feel that the patient’s and family's cultural background might have played a part in this decision, as stigma towards mental illness is a well-documented barrier to depression treatment in the Latino population [12].

For our patient, the mother was considered an acceptable historian and interpreter, and we were unable to communicate directly with the patient most of the time. When we offered to scribe and use the written word, an agreement was not made. It is also important to note that, per the parent, the patient always preferred being roomed with her due to communication deficits. This was challenging to accommodate due to the limited resources. The patient also never presented alternative communication methods, such as sign language or other devices, despite being asked about them during appointments. However, non-verbal behaviors can be used to assess depression in patients, such as looking down, picking or scratching at hair, face, or body, and/or downturned mouth or lowered brows [13]. Being aware of these particular non-verbal cues and other potential non-verbal cues associated with depression can be crucial to those caring for individuals with Goldenhar syndrome.

Given that individuals with Goldenhar syndrome can have a normal lifespan, it is also essential to consider developing a plan to transition them into adulthood. An aspect that is not often considered but is necessary for transition is patient awareness of their disease, as it has been found that many adolescents transitioning to adulthood may not be fully aware of disease-related knowledge [14]. Therefore, the patient must be included in all discussions regarding their health and treatment so they can be more involved in their matters. Timing and education regarding self-management are essential components of the transition. They must be determined using the patient’s individual needs as well as the readiness of the supporting family [15]. The patient, the healthcare team, and supporting family members all need to be involved in the transition plan (Table 1).

**Table 1:** Aspects of transition into adult care for adolescent patients with chronic illnesses This table summarizes key considerations in the transition from pediatric to adult care for adolescents with chronic illnesses, focusing on patient education, support system involvement, and medical team responsibilities [14,15]. Table created by: Authors.

Patient Education	Support System Education	Medical Team Involvement
The patient should have knowledge of their illness, medications and their purposes, and any information regarding advanced directives. The patient should also be included in the transition planning. Patient familiarity with medical units and facilities where care will be provided and involvement in peer support groups can help the patient develop a sense of reassurance and ease the transition process.	Support system members such as caregivers and close relatives should educate patients about their illnesses and medications. The support system should involve the patient in transition planning and preparation. This includes encouraging the patient to ask questions or seek clarification.	The medical team should ensure that the patient has awareness of their illness and the importance of complying with medications. This can include having the patient explain what they know about their illness and medication purposes. The medical team should aim to familiarize the patient with the medical facilities where the patient will obtain care. The medical team should seek involvement in training courses to help develop strong communication skills and improve medical knowledge.

## Conclusions

This case highlights the importance of recognizing the mental health impact of chronic conditions such as Goldenhar syndrome, particularly in patients with communication deficits. Difficulties in establishing consistent mental health support contributed to challenges with preventive care, self-management, and engagement in treatment. Clinicians should remain attentive to these barriers and incorporate mental health care as a core component of complex medical management. This includes timely detection and finding ways for screenings to be applicable based on the patient’s ability to answer and discuss. Options for communication through alternative methods should also be further explored and added to the therapy plan. Lastly, a productive partnership with the families of children with complex health needs can help secure prompt preventive care, support, and treatment when necessary.
